# Diagnosis and management of tropomyosin receptor kinase (TRK) fusion sarcomas: expert recommendations from the World Sarcoma Network

**DOI:** 10.1016/j.annonc.2020.08.2232

**Published:** 2020-09-03

**Authors:** G. D. Demetri, C. R. Antonescu, B. Bjerkehagen, J. V. M. G. Bovée, K. Boye, M. Chacón, A. P. Dei Tos, J. Desai, J. A. Fletcher, H. Gelderblom, S. George, A. Gronchi, R. L. Haas, N. Hindi, P. Hohenberger, H. Joensuu, R. L. Jones, I. Judson, Y.-K. Kang, A. Kawai, A. J. Lazar, A. Le Cesne, R. Maestro, R. G. Maki, J. Martín, S. Patel, F. Penault-Llorca, C. Premanand Raut, P. Rutkowski, A. Safwat, M. Sbaraglia, I.-M. Schaefer, L. Shen, C. Serrano, P. Schöffski, S. Stacchiotti, K. Sundby Hall, W. D. Tap, D. M. Thomas, J. Trent, C. Valverde, W. T. A. van der Graaf, M. von Mehren, A. Wagner, E. Wardelmann, Y. Naito, J. Zalcberg, J.-Y. Blay

**Affiliations:** 1 Dana-Farber Cancer Institute and Ludwig Center at Harvard Medical School, Boston; 2 Department of Pathology, Memorial Sloan Kettering Cancer Center, New York, USA; 3 Department of Pathology, Oslo University Hospital, Oslo, Norway; 4 Department of Pathology, Leiden University Medical Center, Leiden, The Netherlands; 5 Department of Oncology, Oslo University Hospital, Oslo, Norway; 6 Oncology Service Chair, Instituto Alexander Fleming, Buenos Aires, Argentina; 7 Department of Pathology, University of Padua, Padova, Italy; 8 Peter MacCallum Cancer Centre, University of Melbourne, Melbourne, Australia; 9 Department of Pathology, Brigham and Women’s Hospital and Harvard Medical School, Boston, USA; 10 Department of Medical Oncology, Leiden University Medical Centre, Leiden, The Netherlands; 11 Medical Oncology, Dana-Farber Cancer Institute, Boston, USA; 12 Department of Surgery, Fondazione IRCCS Istituto Nazionale Tumori, Milan, Italy; 13 Department of Radiotherapy, Netherlands Cancer Institute, Amsterdam, The Netherlands; 14 Institute of Biomedicine of Sevilla (IBIS, HUVR, CSIC, Universidad de Sevilla), Sevilla; 15 Medical Oncology Department, University Hospital Virgen del Rocio, Sevilla, Spain; 16 Division of Surgical Oncology and Thoracic Surgery, Mannheim University Medical Center, Mannheim, Germany; 17 Department of Oncology, Helsinki University Hospital and University of Helsinki, Helsinki, Finland; 18 Sarcoma Unit, Royal Marsden NHS Foundation Trust, London; 19 Division of Clinical Studies, Institute of Cancer Research, London, UK; 20 Department of Oncology, University of Ulsan College of Medicine, Seoul, Korea; 21 Department of Musculoskeletal Oncology, National Cancer Center, Tokyo, Japan; 22 Pathology, The University of Texas MD Anderson Cancer Center, Houston, USA; 23 Medical Oncology, Insitut Gustave Roussy, Villejuif, Ile-de-France, France; 24 Unit of Oncogenetics and Functional Oncogenomics, Centro di Riferimento Oncologico di Aviano (CRO Aviano) IRCCS, National Cancer Institute, Aviano, Italy; 25 Abramson Cancer Center, Perelman School of Medicine, University of Pennsylvania, Philadelphia, USA; 26 Department of Sarcoma Medical Oncology, Division of Cancer Medicine, The University of Texas MD Anderson Cancer Center, Houston, USA; 27 Centre Jean Perrin, Clermont-Ferrand, France; 28 Division of Surgical Oncology, Brigham and Women’s Hospital, Center for Sarcoma and Bone Oncology, Dana-Farber Cancer Institute, Harvard Medical School, Boston, USA; 29 Department of Soft Tissue/Bone Sarcoma and Melanoma, Maria Sklodowska-Curie National Research Institute of Oncology, Warsaw, Poland; 30 Department of Oncology, Aarhus University Hospital, Aarhus, Denmark; 31 Department of GI Oncology, Peking University Cancer Hospital and Institute, Beijing, China; 32 Sarcoma Translational Research Program, Vall d’Hebron Institute of Oncology, Barcelona; 33 Medical Oncology Department, Vall d’Hebron Hospital, Barcelona, Spain; 34 Department of General Medical Oncology, Leuven Cancer Institute, University Hospitals Leuven, Leuven, Belgium; 35 Cancer Medicine Department, Fondazione IRCCS Istituto Nazionale Tumori, Milan, Italy; 36 Department of Oncology, The Norwegian Radium Hospital, Oslo University Hospital, Oslo, Norway; 37 Memorial Sloan Kettering Cancer Center and Weill Cornell Medical College, New York, USA; 38 The Kinghorn Cancer Centre and Cancer Theme, Garvan Institute of Medical Research, Darlinghurst, Australia; 39 Sylvester Comprehensive Cancer Center at University of Miami Miller School of Medicine, Miami, USA; 40 Department of Medical Oncology, Netherlands Cancer Institute, Amsterdam; 41 Department of Medical Oncology, Radboud University Medical Centre, Nijmegen, The Netherlands; 42 Department of Hematology and Medical Oncology, Fox Chase Cancer Center, Philadelphia; 43 Dana-Farber Cancer Institute and Harvard Medical School, Boston, USA; 44 Gerhard Domagk Institute of Pathology, University of Münster, Münster, Germany; 45 National Cancer Center Hospital East, Kashiwa, Japan; 46 Department of Epidemiology and Preventative Medicine, School of Public Health and Preventive Medicine, Monash University, Melbourne; 47 Department of Medical Oncology, Alfred Health, Melbourne, Australia; 48 Centre Léon Bérard, Unicancer, LYRICAN and Université Claude Bernard Lyon 1, Lyon, France

**Keywords:** entrectinib, larotrectinib, neurotrophic tyrosine receptor kinase, sarcoma, tropomyosin receptor kinase

## Abstract

Sarcomas are a heterogeneous group of malignancies with mesenchymal lineage differentiation. The discovery of neurotrophic tyrosine receptor kinase (*NTRK*) gene fusions as tissue-agnostic oncogenic drivers has led to new personalized therapies for a subset of patients with sarcoma in the form of tropomyosin receptor kinase (TRK) inhibitors. *NTRK* gene rearrangements and fusion transcripts can be detected with different molecular pathology techniques, while TRK protein expression can be demonstrated with immunohistochemistry. The rarity and diagnostic complexity of *NTRK* gene fusions raise a number of questions and challenges for clinicians. To address these challenges, the World Sarcoma Network convened two meetings of expert adult oncologists and pathologists and subsequently developed this article to provide practical guidance on the management of patients with sarcoma harboring *NTRK* gene fusions. We propose a diagnostic strategy that considers disease stage and histologic and molecular subtypes to facilitate routine testing for TRK expression and subsequent testing for *NTRK* gene fusions.

## INTRODUCTION

Sarcomas are a heterogeneous group of malignancies that exhibit mesenchymal lineage differentiation. They arise in either soft tissue (~80%) or bone (~20%) and comprise ~70 malignant subtypes (per World Health Organization classification), each with distinct underlying biology and clinical behavior.^1^ Sarcomas account for approximately 1% of all adult cancers and 20% of all pediatric solid tumors.^2^ Complete resection (with or without radiation and/or chemotherapy) forms the mainstay of curative management for most subtypes in the localized setting. For patients diagnosed with locally advanced or metastatic disease, or those with disease recurrence following surgery, treatment options include systemic therapy and potential local approaches such as radiation, isolated limb perfusion, surgery, and ablation techniques.^3^ The median overall survival of patients with advanced soft tissue sarcomas is approximately 20 months, with most patients deriving only transient benefit from palliative chemotherapy.^4,5^ Therefore, there is a clear unmet need for more effective systemic therapies for patients with advanced/metastatic sarcomas.

As our understanding of the molecular basis of tumorigenesis has improved with advances in diagnostic technology, precision oncology approaches to the treatment of sarcomas have emerged. A classic example of this is mutational profiling of *KIT*, *PDGFRA*, and other genes to predict sensitivity of gastrointestinal stromal tumors (GISTs) to imatinib and other KIT/PDGFRA tyrosine kinase inhibitors.^6–11^ More recently, the discovery of neurotrophic tyrosine receptor kinase (*NTRK*) gene fusions as pan-tumor oncogenic drivers has provided new precision medicine-based treatment options for a subset of patients with sarcoma.^12^ The rarity and diagnostic complexity of this particular biomarker raise a number of questions and challenges for clinicians.

To address these issues, the World Sarcoma Network, a think tank gathering national and international sarcoma groups for the past 10 years, convened two consensus meetings of expert adult oncologists and pathologists to discuss diagnostic challenges and propose a diagnostic strategy in this area. We subsequently developed this article to provide practical guidance on how to optimally integrate the *NTRK* gene fusion biomarker into the clinical management of patients with sarcoma.

## *NTRK* GENE FUSIONS

The *NTRK* genes *NTRK1* (chromosome 1q23.1), *NTRK2* (chromosome 9q21.33), and *NTRK3* (chromosome 15q25.3) are typically involved in normal neuronal development and encode the tropomyosin receptor kinase (TRK) proteins, traditionally known as TRKA, TRKB, and TRKC, respectively.^12,13^ Wild-type TRK proteins are activated through oligomerization mediated by the binding of neurotrophin ligands.^14^ Subsequent downstream signaling contributes to central nervous system (CNS) development and regulation of appetite, body weight, memory, mood, movement, pain, and proprioception.^15–20^

*NTRK* gene fusions have been identified in a diverse range of adult and pediatric tumor types.^12^ These fusions result from inter- or intra-chromosomal rearrangements leading to juxtaposition of the 3′ region of an *NTRK* gene (encoding the full kinase domain) with the 5′ region of a partner gene (encoding an oligomerization or other protein-association domain), ultimately producing a constitutively active TRK fusion protein.^21^ Fusions involving *NTRK* gene 5^′^ regions have also been reported, although the pathogenicity of these is unclear.^22^ In addition to fusions, *NTRK* gene point mutations and amplifications have been identified in a variety of different cancer types; however, roles for these aberrations in tumorigenesis have not been established.^22^

While rare in most common tumor types (e.g. lung and colorectal cancers), *NTRK* gene fusions are reported to be recurrent in a subset of rare tumor types (e.g. secretory carcinoma of the salivary gland, secretory carcinoma of the breast, congenital mesoblastic nephroma, pediatric melanoma, and infantile fibrosarcoma).^21,23,24^ One of the first discovered and most well characterized fusions, *ETV6-NTRK3*, resulting from a t(12;15)(p13;q25) translocation, is present in >90% of infantile fibrosarcomas.^25,26^ By contrast, *NTRK* fusions have been identified in other adult and pediatric sarcomas at a frequency of <1%.^23,27,28^ Recent studies investigating *NTRK* fusions among mesenchymal neoplasms have identified a number of emerging new soft tissue tumor entities displaying various phenotypes, which resemble lipofibromatosis, fibrosarcoma, and malignant peripheral nerve sheath tumors (Table 1). A significant number of these *NTRK* fusion-positive tumors show co-expression of S100 protein and CD34, while the rest have a nonspecific immunophenotype.^27,29–32^ The published literature on *NTRK* gene fusion frequency in sarcomas is limited and more data are needed.

## TARGETED THERAPY FOR TRK FUSION CANCERS

*NTRK* gene fusions (but not other *NTRK* alterations) appear to be primary oncogenic drivers in the tumors that harbor them. The encoded fusion proteins feature constitutive tyrosine kinase activity that may be targeted clinically with a number of agents that are either approved or in development.^12,33^

### Larotrectinib

Larotrectinib is a first-in-class, ATP-competitive, small-molecule inhibitor of TRK. It is highly potent, with IC_50_ values in the range of 6.5–10.6 nM, and highly selective for TRKA, B, and C, with binding affinities over 100-fold greater than for a panel of other kinases.^24,34,35^ Larotrectinib is approved by the US Food and Drug Administration (FDA) and European Medicines Agency (EMA) for use in adult and pediatric patients with solid tumors harboring an *NTRK* gene fusion who have disease that is locally advanced or metastatic, or where surgery is likely to result in severe morbidity, and who have no satisfactory treatment options.^36,37^ Patients with a known resistance mutation are not indicated for larotrectinib treatment.

Larotrectinib has demonstrated robust efficacy in a combined analysis of three phase I/II trials in adults and children with TRK fusion cancers, irrespective of age or tumor type.^38^ In an integrated dataset of 159 patients, investigator-assessed objective response rate (ORR) was 79% [95% confidence interval (CI) 72% to 85%] and median duration of response was 35.2 months (median follow-up 12.9 months). The median time to response was 1.8 months.^39^ Objective responses and durable disease control were also observed in the subsets of patients with primary CNS tumors or non-CNS solid tumors with brain metastases.^40,41^ Larotrectinib-related adverse events of grade 3–4 occurred in 13% of patients, and dose reductions and treatment discontinuations due to treatment-related adverse events occurred in 8% and 2% of patients, respectively.^39^ Long-term follow-up is ongoing. The favorable safety profile of larotrectinib, together with robust clinical efficacy, translated into rapid, sustained, and clinically meaningful improvements in quality of life in the majority of patients.^42^

Among the 17 different tumor types represented in the larotrectinib dataset, the most common (47%) were sarcomas.^39^ Of 71 patients with a sarcoma, two (3%) had an osteosarcoma and a dedifferentiated chondrosarcoma, four (6%) had a GIST, 29 (41%) had infantile fibrosarcoma, and 36 (51%) had other soft tissue tumors, including adult fibrosarcoma, inflammatory myofibroblastic tumor, infantile myofibromatosis, lipofibromatosis, malignant peripheral nerve sheath tumor, myopericytoma, spindle cell sarcoma, high-grade endometrial stromal tumor, and synovial sarcoma.^43^ The histologic subtypes of these patients were captured as reported by the investigators; however, due to the very rare nature of the subtypes reported and the varied nomenclature used in sarcoma pathology, a central pathology review and efficacy analysis by sarcoma subtype is warranted. Furthermore, data on other driver alterations in these patients would be informative.

The ORR with larotrectinib in adult and pediatric patients with sarcoma harboring an *NTRK* fusion was 74% (95% CI 52% to 90%) and 94% (95% CI 82% to 99%), respectively. Objective responses were observed in patients with soft tissue sarcomas, GISTs, and infantile fibrosarcoma. Of two patients with a bone sarcoma, one had a partial response and one had stable disease. At a median follow-up of 15.6, 13.0, and 14.1 months, median duration of response, progression-free survival, and overall survival were not estimable (NE; range 1.6+ to 44.2+), 28.3 (95% CI 16.8-NE), and 44.4 (95% CI 44.4-NE) months, respectively (Table 2). Grade 3–4 adverse events related to larotrectinib were reported in 13% of patients.^43^

### Entrectinib

Entrectinib is a multi-targeted, pan-TRK, ROS1, and ALK inhibitor. It has low to sub-nanomolar enzymatic activity against TRKA, TRKB, TRKC, ROS1, and ALK (IC_50_ values of 1.7, 0.1, 0.1, 0.2, and 1.6 nM, respectively).^44^ Entrectinib is FDA-approved for use in adult and pediatric patients ≥12 years of age with solid tumors harboring an *NTRK* gene fusion who have disease that is metastatic or where surgery is likely to result in severe morbidity, and who have progressed following treatment or have no satisfactory alternative therapy. Patients with a known resistance mutation are not indicated for entrectinib treatment. Entrectinib is also FDA-approved for patients with metastatic non-small cell lung cancer (NSCLC) harboring a *ROS1* gene fusion.^45^

Entrectinib demonstrated tumor-agnostic efficacy in an integrated analysis of 54 patients with TRK fusion cancers in one of three phase I/II trials. Independently assessed ORR was 57% (95% CI 43% to 71%) and median duration of response was 10.4 months (median follow-up 12.9 months). Clinically meaningful and durable intracranial responses were seen in patients with brain metastases. Adverse events with entrectinib were mainly grade 1 or 2 and the proportion of patients with dose reductions and treatment discontinuations due to a treatment-related adverse event was 27% and 4%, respectively.^46^

Among 13 patients with sarcoma in the overall entrectinib clinical trial dataset, six subtypes were identified: cervix adenosarcoma, dedifferentiated chondrosarcoma, endometrial stromal sarcoma, follicular dendritic cell sarcoma, GIST, and malignant peripheral nerve sheath tumor. Of note, there were no patients with infantile fibrosarcoma enrolled in these trials. The ORR for the sarcoma subset was 46%. Median duration of response, progression-free survival, and overall survival were 10.3 (95% CI 4.6–15.0), 11.0 (95% CI 6.5–15.7), and 16.8 (95% CI 10.6–20.9) months, respectively (Table 2).^47^

## METHODS OF *NTRK* GENE FUSION TESTING

*NTRK* gene rearrangements and fusion transcripts can be detected with different molecular pathology techniques such as FISH, reverse transcription polymerase chain reaction (RT-PCR), and massive parallel sequencing (MPS), while TRK protein expression can be demonstrated by immunohistochemistry (IHC) (Table 3).^48,49^

### Fluorescence in situ hybridization

FISH employs fluorescently labeled DNA probes that anneal to specific regions within or flanking a gene(s) of interest. To detect a particular *NTRK* gene fusion, colocalization of FISH probes to each gene component of the fusion can be demonstrated.^27^ In practice, however, it is more feasible to use break-apart FISH probes that flank each of the three *NTRK* genes and demonstrate rearrangement without identifying the fusion partner gene.^27,34^ The efficient break-apart approach avoids the need to develop an unrealistically large number of FISH probe sets for uncommon fusions and also detects novel fusions with as yet uncharacterized fusion partners. FISH is available in many clinical laboratories, has a short turnaround time, and is relatively inexpensive; however, specific expertise is required to interpret test results, particularly in paraffin sections where nuclear slicing can result in artifacts. A false-negative rate of up to 30% has been reported with FISH in pediatric sarcomas.^50^ Furthermore, FISH does not distinguish between in-frame and out-of-frame fusion events.

### Immunohistochemistry

IHC typically employs an antibody that binds to antigens common to the C-terminal domain of all three TRK proteins (pan-TRK IHC) to detect elevated TRK protein expression (Table 4). This method relies on the fact that most normal cells express low levels of TRK while tumor cells harboring an *NTRK* gene fusion typically display elevated TRK protein levels. Nevertheless, TRKA, TRKB, and TRKC expression can be observed by IHC in some normal cells: neurons, myenteric plexus, endothelial cells, and podocytes. In adult tissue, expression is restricted to smooth muscle, testes, and neuronal components. These components can be used as internal (endothelial cells, myenteric plexus) or external (podocytes in renal tissue) positive IHC controls. IHC represents a useful indirect readout for *NTRK* gene rearrangements. However, variable rates of sensitivity (75%−88%) and specificity (81%−96%) have been reported,^51,52^ which may be explained by the use of different antibodies, different IHC detection protocols, and poor or excessive tissue fixation. Any of these variables can impact IHC staining and intensity. The overall positive and negative predictive values in one IHC study have been reported to be 11.2% and 99.8%, respectively (using pan-TRK antibody clone EPR17341).^51^ Moreover, sensitivity has been shown to vary according to the *NTRK* gene involved, with lower sensitivity reported for *NTRK3* (55%−79%) compared with *NTRK1* (88%−96%) and *NTRK2* (89%−100%).^51,52^ A TRK IHC signal should be considered as positive when staining of ≥1% of tumor cells is observed.^53^ Furthermore, the subcellular pattern of pan-TRK IHC staining may indicate the nature of the underlying gene fusion, with nuclear staining suggestive of *NTRK3* fusions and moderate to strong, diffuse cytoplasmic staining suggestive of *NTRK1*/*NTRK2* fusions.^49,53,54^

One benefit of IHC over molecular analyses is that it provides evidence of the expression of the protein target of TRK inhibitors. Of six patients with primary resistance to larotrectinib among the initial 55 patients treated in clinical trials, three had tumor material available for central analysis and in all three cases, pan-TRK IHC did not demonstrate elevated TRK protein expression, indicating that the rearrangements detected by molecular testing did not yield chimeric proteins with an intact TRK C-terminus in these cases. One additional patient harbored an *NTRK3* kinase domain mutation that conferred resistance.^38^

**Figure 1.**
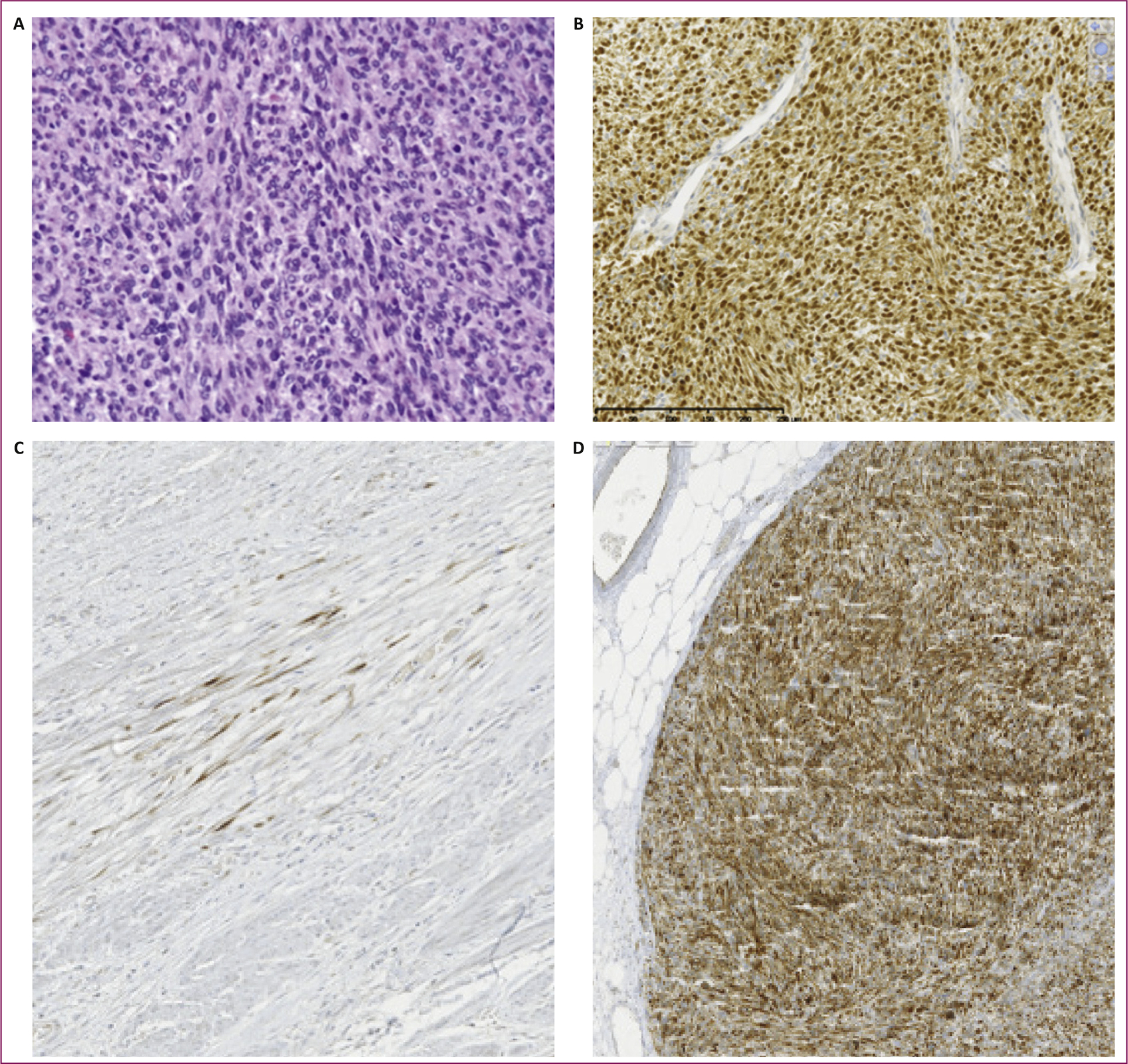
Examples of positive TRK IHC staining. (A) *ETV6-NTRK3* fusion infantile fibrosarcoma stained by hematoxylin and eosin and (B) pan-TRK IHC with A7H6R clone (Cell Signaling Technology) and ultraView detection. (C) Focal staining in a leiomyosarcoma without *NTRK* gene alterations. (D) Intense staining in a leiomyosarcoma with *NTRK1* copy number gain. IHC, immunohistochemistry; *NTRK*, neurotrophic tyrosine receptor kinase receptor; TRK, tropomyosin receptor kinase.

IHC is widely available in clinical laboratories, allows a rapid turnaround time, and is far less expensive than FISH. Although more data on sensitivity and specificity are needed, pan-TRK IHC is considered to have a false-negative rate of ~10%.^52^ In a study of seven patients with soft tissue spindle cell tumors and *NTRK3* rearrangement detected by both IHC and FISH, rearrangements were only confirmed by RNA sequencing in three cases.^30^ Therefore, sarcomas with a high probability of harboring an *NTRK* fusion but with negative pan-TRK IHC staining should be considered for confirmatory testing with a genomic method (FISH or MPS). IHC may prove to be a valuable screening tool to highlight *NTRK* rearrangements, with the exception of CNS and neuroendocrine tumors where IHC is not a reliable screening tool because of endogenous elevated TRK expression. Furthermore, many sarcomas with myogenic or neural differentiation may display focal TRK expression.^53^ Thus, in these tumors, only diffuse staining should be considered positive. Strong TRK IHC staining can also be found in cases with *NTRK* gene amplification, supporting the requirement to confirm the presence of *NTRK* rearrangements with an orthogonal molecular method (Figure 1).^30^

### Reverse transcription polymerase chain reaction

RT-PCR uses primers flanking the breakpoint region in the transcript encoded by the fused genes, with the 3′ primer annealing to an *NTRK* gene and the more 5′ primer annealing to the relevant fusion partner gene. When present in the tumor, the targeted portion of the fusion transcript will be amplified yielding a positive RT-PCR result. RT-PCR is a widely established technique and is rapid and inexpensive. Multiplex RT-PCR can be carried out using primer sets specific to a number of known *NTRK* fusion genes in a single assay. However, this method does not detect *NTRK* gene fusions with unknown partner genes; thus, a negative RT-PCR result cannot exclude the presence of a fusion. Therefore, RT-PCR is only recommended in settings where a specific type of fusion is expected following histologic-based triage, for instance, *ETV6-NTRK3* in infantile fibrosarcoma, or as a complementary method for gene rearrangements detected by FISH.

### Massive parallel sequencing

MPS allows for the simultaneous detection of fusions between *NTRK1*—*3* and any number of fusion partner genes, depending upon the particular assay used. Targeted MPS with a panel of primers that hybridize to select regions in predefined genes is the preferred method. DNA-based MPS assays are not the best approach for identifying all *NTRK* fusions, especially those involving the *NTRK2* and *NTRK3* genes because of their large introns. Targeted RNA MPS allows for more systematic detection of *NTRK* fusion transcripts. Adequately designed targeted RNA MPS panels allow for the detection of novel *NTRK* gene fusion partners, and there are a number of commercially available assays that cover all three *NTRK* genes. However, it should be noted that different commercial panels have shown some remarkable differences in detection rate.^55^ MPS is not routinely conducted in all clinical laboratories, has a relatively long turnaround time, and is quite expensive, particularly if only a limited number of tests are required. However, various groups are continuing to develop RNA MPS platforms that can detect in parallel the multitude of fusion genes observed in sarcomas.

In addition to functional *NTRK* fusion transcripts, RNA-based MPS assays may identify non-oncogenic aberrant *NTRK* rearrangements (incidental genomic alterations) that do not yield constitutively active fusion proteins. Clinicians should be aware of this possibility and understand how to interpret complex MPS data reports. If expression of the fusion protein is in doubt, IHC may be a useful confirmatory tool. Similarly, *NTRK* point mutations occur more often than gene fusions and may also be identified by MPS assays; however, these mutations are not considered predictive of treatment response.^56^

## TESTING FOR *NTRK* GENE FUSIONS IN SARCOMAS

Given the robust efficacy and favorable safety profiles of TRK inhibitors demonstrated in patients with TRK fusion sarcomas, testing for *NTRK* gene fusions should be incorporated into the clinical management of patients with sarcoma, with prioritization in specific stages and subtypes, as discussed below. The rarity of these oncogenic drivers presents a number of challenges, including the cost of testing, limited resources, limited tumor tissue, and the complexities of integrating a new molecular test into the current diagnostic workup. However, the overall benefit of molecular testing in the diagnosis and clinical management of patients with sarcoma has been demonstrated in large multicenter studies, similar to what has been shown for lung cancer.^57,58^ While sequence-based testing methods (RNA MPS or RT-PCR) are recommended for the detection of productive *NTRK* gene rearrangements, IHC with a validated antibody against TRK proteins (most easily with a pan-TRK antibody) may be used as a fast and less expensive pre-screening tool. Furthermore, selecting histotypes negative for pathognomonic genetic alterations (other translocations, kinase mutations, *MDM2*/*CDK4* amplification) could allow exclusion of ~45% of all sarcomas from *NTRK* gene fusion testing, given the mutual exclusivity of such driver alterations.^59,60^

*NTRK* fusion testing may be prioritized in disease settings where TRK inhibitor therapy is most relevant, also considering that some of the recently reported *NTRK*-rearranged entities tend to behave indolently. The majority of sarcoma patients are diagnosed while still localized and these tumors are amenable to curative surgical resection without the need for systemic therapy. Therefore, *NTRK* fusion testing in primary, resectable sarcomas may not be necessary (except when used for definitive diagnosis, such as in the case of putative infantile fibrosarcomas). Nevertheless, for patients at high risk of relapse, *NTRK* gene fusion testing might provide clinically actionable information for later in the disease course. Testing for *NTRK* gene fusions should be carried out in patients with locally advanced, unresectable tumors or in those with metastatic disease failing conventional therapies.

### Sarcomas with a high NTRK fusion frequency (priority 1)

**Figure 2.**
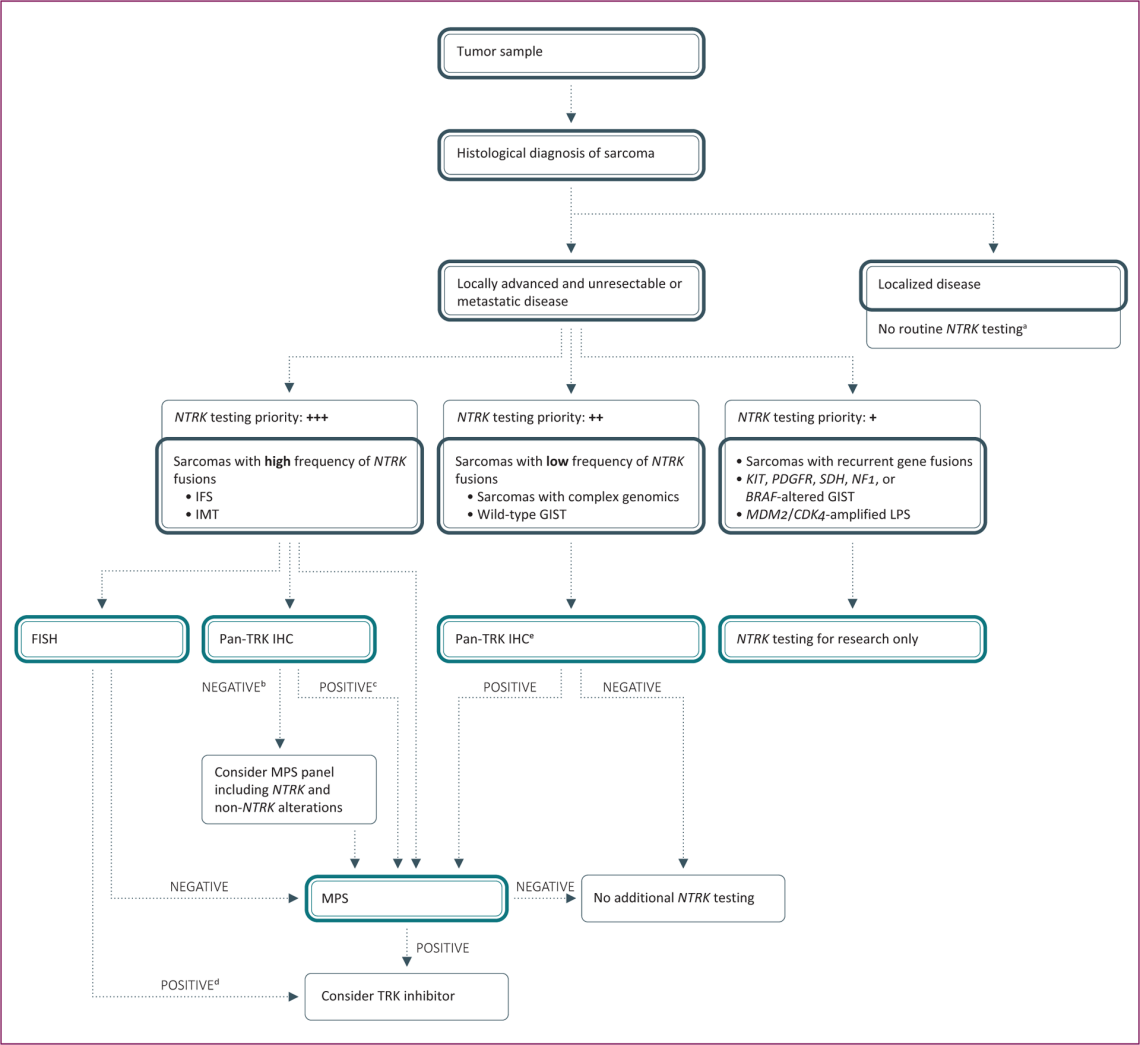
Recommended algorithm for *NTRK* gene fusion testing in sarcomas. GIST, gastrointestinal stromal tumor; IFS, infantile fibrosarcoma; IHC, immunohistochemistry; IMT, inflammatory myofibroblastic tumor; LPS, liposarcoma; MPS, massive parallel sequencing; *NTRK*, neurotrophic tyrosine receptor kinase; RT-PCR, reverse transcription polymerase chain reaction; TRK, tropomyosin receptor kinase. ^a^ For patients at high risk of relapse, *NTRK* gene fusion testing might provide clinically actionable information for later in the disease course. ^b^ If histology is typical then confirmation by MPS is recommended. ^c^ Treatment may be considered concurrently with confirmatory MPS. ^d^ Consider parallel validation by MPS or RT-PCR to confirm that fusion is in-frame. ^e^ Avoid IHC screening in cases with myogenic and neural differentiation due to the high rate of false positivity.

Given the potential cost and resource limitations of universal testing, we propose a three-tiered diagnostic algorithm for the prioritization of *NTRK* gene fusion testing according to the likelihood of finding a fusion (Figure 2). The highest priority for *NTRK* fusion testing is given to the histologic subtypes that commonly or non-infrequently harbor *NTRK* gene fusions, such as infantile fibrosarcomas^61^ and *ALK* and *ROS1* fusion-negative inflammatory myofibroblastic tumors.^62^ These entities should be tested upfront for *NTRK* fusions in all situations^53^ and the test should ideally be ordered by the pathologist following central pathologic diagnosis. In fact, *NTRK* fusion testing is often conducted as part of the diagnostic process for suspected infantile fibrosarcomas. For histologic subtypes with a high pre-test probability of harboring an *NTRK* fusion, we recommend the use of FISH, IHC, or MPS. A negative FISH result should be confirmed by MPS. For a positive FISH result, confirmation that the fusion is in-frame by MPS or RT-PCR should be considered in parallel to treatment. MPS confirmation of a negative IHC result is recommended for cases with typical histology. For cases with positive IHC results, treatment may be considered concurrently with confirmatory MPS.

### Sarcomas with a low NTRK fusion frequency (priority 2)

*NTRK* gene fusions are thought essentially to be mutually exclusive to other primary oncogenic drivers.^59^ In a study of patients with various tumor types, 31% of *NTRK* fusion-negative cases harbored activating MAPK pathway alterations compared with only 1.5% (*n* = 1) of *NTRK* fusion-positive cases.^60^ In another study, among 103 sarcomas tested for recurrent kinase fusions in one study, one sample had an *NTRK1* gene fusion but no other concurrent fusions.^63^ Therefore, for sarcoma subtypes where *NTRK* gene fusions are rare, *NTRK* fusion screening should only be routinely done in cases already known to lack canonical oncogene alterations, such as wild-type GISTs and sarcomas with complex genomics. Sarcomas with recurrent gene fusions, GISTs with *KIT*, *PDGFR*, *SDH*, *NF1*, or *BRAF* alterations, and liposarcomas with *MDM2* or *CDK4* amplification may be excluded from routine *NTRK* fusion testing. Of this subset, however, tumors that do not show specific lineage differentiation (i.e. positive only for vimentin) may be enriched in molecular alterations including *NTRK* fusions.

### Sarcomas with canonical oncogene alterations (priority 3)

Very infrequent situations of *NTRK* gene fusions co-occurring with other driver alterations in untreated tumors have been reported; however, the *NTRK* fusion appears to exert oncogenic dominance in these rare cases.^59,60^ Therefore, *NTRK* fusion testing in tumors with canonical pathognomonic alterations may be valuable in a research context in order to provide data on frequency and clinical significance of co-occurring *NTRK* gene fusions. While *NTRK* gene fusions have been identified in a range of sarcoma subtypes (Table 1), comprehensive data on *NTRK* fusion frequency in different sarcoma subtypes are lacking. Therefore, the majority of soft tissue and bone sarcomas should continue to be studied until there are sufficient data to guide future diagnostic approaches. Comprehensive data about *NTRK* gene fusion frequency in different sarcoma subtypes and correlation with morphological features would better inform the optimal approach to *NTRK* gene fusion screening in sarcomas and should be collected. In this regard, a prospective registry and retrospective collection of TRK fusion sarcoma cases would be valuable and a study is planned in Spain and France where all soft tissue sarcomas will be prospectively screened with pan-TRK IHC, with positive cases then confirmed by MPS.

## CLINICAL MANAGEMENT OF TRK FUSION SARCOMAS

The labeled indications for larotrectinib (FDA and EMA) and entrectinib (FDA) include patients for whom surgery is likely to result in severe morbidity or who have no satisfactory alternative therapy. Therefore, the advantages and disadvantages of TRK inhibitors compared with other available therapies should be discussed by the patient and the treating physician.

In addition to the overall efficacy of TRK inhibitors described earlier (Table 2), efficacy of neoadjuvant larotrectinib therapy has also been demonstrated in situations where surgery would otherwise result in life-changing operations (e.g. amputation). Five children with locally advanced TRK fusion sarcomas (three with infantile fibrosarcomas and two with other soft tissue sarcomas) achieved a partial response to neoadjuvant larotrectinib and underwent resection after a median of six treatment cycles. Resections were R0 (negative resection margins with no tumor at the inked resection margin) in three patients, R1 (microscopic residual tumor at the resection margin) in one patient and R2 (incomplete resection with macroscopic residual tumor) in one patient. Three patients achieved complete or near-complete pathological responses and at last follow-up remained disease-free 7–15 months after surgery.^64^ While these data are encouraging, the question of if and when to discontinue TRK inhibitor therapy following a complete response still remains.

For patients with metastatic disease requiring systemic therapy, treatment with larotrectinib or entrectinib is approved after failure of standard therapies and may be valuable after standard first-line treatment given the rapid, durable responses and tolerability observed. In clinical trials of larotrectinib and entrectinib, responses were typically observed at the time of the first protocol-mandated tumor assessment, and pseudo-progression is uncommon with these therapies; therefore, it may be possible to quickly evaluate treatment response. However, it should be noted that no data exist for larotrectinib or entrectinib compared or combined with standard systemic cytotoxic therapies. Furthermore, the long-term safety profile of TRK inhibitors remains unknown and requires further study.

*NTRK* gene fusions have been shown to persist in tumors over time,^60^ suggesting that they remain the dominant oncogenic driver over the course of different treatments. This provides the rationale for a sequential TRK inhibitor treatment approach in patients with TRK fusion cancer, similar to current practice in oncogene-addicted (e.g. EGFR, ALK) NSCLC. The next-generation TRK inhibitors selitrectinib and repotrectinib have shown encouraging activity in patients who had progressed on larotrectinib or entrectinib due to acquired resistance mutations in the TRK kinase domain, including patients with sarcoma.^65–67^

## SUMMARY

The emergence of *NTRK* gene fusions as clinically actionable biomarkers marks a new era in precision oncology, with the tumor-agnostic approvals of larotrectinib and entrectinib representing milestones in drug development. TRK inhibitors provide new personalized treatment options with the potential to extend survival and improve quality of life in some patients with sarcoma harboring *NTRK* gene fusions. Integrating *NTRK* fusion testing into the current diagnostic workup of patients with sarcoma is particularly challenging due to the rarity of this biomarker. Here, we propose a diagnostic strategy to address this that considers disease stage and histologic and molecular subtypes to facilitate routine testing for TRK expression and subsequent testing for *NTRK* gene fusions.

Routine genome-wide MPS in sarcomas may not currently be cost-effective due to the small number of additional genomic alterations to be tested. However, IHC provides a valuable pre-screening tool and focused MPS panels, such as those that detect *NTRK* gene fusions and other key gene fusions in parallel, are particularly relevant for sarcomas. Further research is necessary to fully establish the sensitivity and specificity of pan-TRK IHC. Furthermore, multinational comparative studies are encouraged to increase the reproducibility of MPS assays. Finally, prospective studies will be essential to determine the frequency of *NTRK* gene fusions in different sarcoma subtypes and correlation with morphological, biological, and clinical features in order to better inform the optimal approach to *NTRK* gene fusion screening.

## Figures and Tables

**Table 1.** Frequency of *NTRK* gene fusions identified in sarcomas

Study	Testing method	Proportion of *NTRK* fusions identified	*NTRK* fusion-positive sarcoma subtypes	*NTRK* genes and fusion partners involved
Agaram et al.^29^	FISH, RNA MPS	71% (10/14)	Lipofibromatosis-like neural tumor	1 *TPR-NTRK1* 1 *TPM3-NTRK1* 4 *LMNA-NTRK1*
Bourgeois et al.^68^	RT-PCR	91% (10/11)	IFS	*ETV6-NTRK3*
Bui et al.^69^	Targeted DNA MPS	0.7% (1/152)	Myopericytoma	NR
Chang et al.^70^	Targeted RNA MPS	33% (3/9)	IMT	*ETV6-NTRK3*
Chmielecki et al.^71^	Targeted RNA MPS	1% (4/324)	IFS (*n* = 2), assorted soft tissue sarcoma (*n* = 1), hemangioma (*n* = 1), bone sarcoma (*n* = 1)	*SQSTM1-NTRK1 (n* = 1), other fusions partners NR
Church et al.^72^	FISH	96% (25/26)	IFS	*NTRK3*
Croce et al.^73^	Targeted RNA MPS	54% (7/13)	Uterine and vaginal sarcomas resembling fibrosarcoma	6 *TPM3-NTRK1,* 1 *EML4-NTRK3*
Gatalica et al.^51^	Targeted RNA MPS	0.4% (2/478)	1 STS (poorly differentiated sarcoma with possible myofibroblastic differentiation), 1 uterine sarcoma (intermediate to high-grade sarcoma of uterine origin, with myxoid stroma and no specific line of differentiation)	1 *TPM3-NTRK1,* 1 *SPECC1L-NTRK3*
Rosen et al.^60^	Targeted RNA MPS	1% (11/944)	Sarcoma NOS [9/770 (1%)], uterine sarcoma [2/174 (1%)]	NR
Shi et al.^74^	Targeted DNA MPS	0.5% (1/186) overall [4% (1/24) in quad-negative tumors]	GIST	*ETV6*-*NTRK3*
Solomon et al.^52^	Targeted DNA and/or RNA MPS	0.7% (13/1915)	IFS (*n* = 2), lipofibromatosis-like neural tumor (*n* = 2), uterine sarcoma (*n* = 2), uterine high-grade pleomorphic sarcoma, high-grade spindle cell sarcoma, malignant spindle cell sarcoma, spindle cell sarcoma, angiosarcoma, S-100 positive malignant spindle cell neoplasm, low grade sarcoma (all *n* = 1)	*LMNA*-*NTRK1* (*n* = 4), *TPM3*-*NTRK1* (*n* = 3), *ETV6-NTRK3 (n* = 2), *TPR-NTRK1, TPM4-NTRK3, EEF1A1-NTRK3, PEAR1-NTRK1* (all *n* = 1)
		18% (3/17)	IMT	*ETV6-NTRK3*
Stransky et al.^63^	TCGA RNA-seq dataset	1% (1/103)	Sarcoma	*TPM3-NTRK1*
Surrey et al.^75^	Targeted RNA MPS	4% (2/45)	Sarcomas (other)	1 *TFG-NTRK3,* 1 *RBPMS-NTRK3*
Suurmeijer et al.^31^	FISH, targeted RNA MPS	60% (15/25)	Malignant peripheral nerve sheath tumor-like	8 *LMNA-NTRK1,* 3 *TPM3-NTRK1,* 1 *SPECC1L-NTRK2,* 1 *TPR-NTRK1,* 2 *NTRK1* with unknown fusion partners
Yamamoto et al.^76^	MPS (TBC), IHC	5% (2/40)	IMT	*ETV6-NTRK3*
Zhu et al.^77^	Targeted RNA MPS	3% (5/184)	Lipofibromatosis-like neural tumor (*n* = 2), IFS (*n* = 1), IMT (*n* = 1), sarcoma NOS (*n* = 1)	2 *ETV6*-*NTRK3* 2 *TPM3-NTRK1* 1 *LMNA*-*NTRK1*

GIST, gastrointestinal stromal tumor; IFS, infantile fibrosarcoma; IHC, immunohistochemistry; IMT, inflammatory myofibroblastic tumor; LPS, liposarcoma; MPS, massive parallel sequencing; NOS, not otherwise specified; NR, not reported; RT-PCR, reverse transcription polymerase chain reaction; STS, soft tissue sarcoma; TCGA, The Cancer Genome Atlas.

**Table 2.** Efficacy of TRK inhibitors in patients with sarcoma harboring *NTRK* gene fusions^a^

	Larotrectinib *n* = 71^43^	Entrectinib *n* = 13^47^
Objective response rate, %	87 (95% CI 77–94)	46 (95% CI 19–75)
Median duration of response, months	NE (range 1.6+ to 44.2+)	10.3 (95% CI 4.6–15.0)
Median progression-free survival, months	28.3 (95% CI 16.8-NE)	11.0 (95% CI 6.5–15.7)
Median overall survival, months	44.4 (95% CI 44.4-NE)	16.8 (95% CI 10.6–20.9)

CI, confidence interval; MPS, massive parallel sequencing; NE, not estimable; *NTRK*, neurotrophic tyrosine receptor kinase receptor; PCR, polymerase chain reaction; TRK, tropomyosin receptor kinase.

a In the larotrectinib clinical trials, *NTRK* gene fusions were detected by local MPS, according to the procedures and analytic pipelines established by each laboratory, or by FISH. All tests were carried out in a Clinical Laboratory Improvement Amendments-certified (or equivalent) laboratory. In the entrectinib clinical trials, *NTRK* gene fusions were detected by central RNA MPS (Trailblaze Pharos, Ignyta, San Diego, CA, USA) or local molecular testing (FISH, quantitative PCR, or DNA or RNA MPS).

**Table 3.** Methods of *NTRK* gene fusion testing

	FISH	IHC	RT-PCR	MPS
Advantages	Available in many clinical laboratories	Widely available	Widely available	Allows simultaneous detection of fusions between *NTRK1—3* and any number of known or novel fusion partner genes
	Rapid turnaround time	Rapid turnaround time	Rapid turnaround time	
	Relatively inexpensive	Inexpensive	nexpensive	
		Allows identification of specific cell types harboring the *NTRK* fusion		
Disadvantages	Requires specific expertise	Detection of wild-type protein expression, especially in tumors with neural and myogenic differentiation	Requires knowledge of fusion partner gene sequence	Not routinely conducted in all clinical laboratories
	False negativity rate of ≤30%	False negativity rate of ~10%; this rate may be higher in tumors harboring *NTRK3* fusions	Challenging to test for fusions involving multiple *NTRK* and fusion partner genes in parallel	Relatively long turnaround time
	Does not distinguish between in-frame and out-of-frame fusion events			Relatively expensive
				DNA MPS may miss *NTRK2* and *NTRK3* fusions due to large introns RNA MPS requires high quality RNA
				Variable detection rates of different panels
				May identify non-oncogenic *NTRK* rearrangements

MPS, massive parallel sequencing; *NTRK*, neurotrophic tyrosine receptor kinase receptor.

**Table 4.** Commercially available pan-TRK antibodies

Clone	Company	Label	Studies
EPR17341	Abcam	RUO	Hechtman et al.^78^ Leica Bond 3 (ER2 retrieval, dilution 6 μg/ml) Solomon et al.^52^ Leica Bond 3 (ER2 retrieval, dilution 6 μg/ml) Gatalica et al.^51^ Ventana Benchmark or Dako Autostainer Rudzinski et al.^79^ Ventana Benchmark (CC1 retrieval, 1:500 dilution) Hung et al.^80^ Manual (1/300 dilution) Xu et al.^81^ Leica Bond 3
EPR17341 (prediluted)	Ventana	CE/IVD	Feng et al.^82^ Ventana assay on dedicated platform
EPR18413	Abcam	RUO	Not reported
C17F1	Cell Signaling Technology	RUO	Murphy et al.^83^ Dako Autostainer (dilution 1:25, cocktail with anti-ALK and anti-ROS1 antibodies)
A7H6R	Cell Signaling Technology	RUO	Not reported

All antibodies are rabbit monoclonal and bind to the TRK C-terminal domain.

IVD, *in vitro* diagnostic; TRK, tropomyosin receptor kinase; RUO, research use only.
